# Cucurbitacin IIb alleviates colitis via regulating gut microbial composition and metabolites

**DOI:** 10.1016/j.heliyon.2024.e38051

**Published:** 2024-09-18

**Authors:** Yinyin Zhao, Kangxiao Guo, Yongwang Yan, Binyuan Jiang

**Affiliations:** a Ningbo Institute of Innovation for Combined Medicine and Engineering (NIIME), The Affiliated LiHuiLi Hospital of Ningbo University, Ningbo, 315000, China; b Pharmaceutical College, Changsha Health Vocational College, Changsha, 410699, China; c Medical Research Center, The Affiliated Changsha Central Hospital, Hengyang Medical School, University of South China, Changsha, 410004, China

**Keywords:** Cucurbitacins, Colitis, Inflammation, Metabolites, Microbiota

## Abstract

Cucurbitacin IIb, a member of the triterpenoid family, exerts beneficial effects on intestinal diseases, including enteritis and bacillary dysentery. However, its effects and mechanisms of action on colitis have not yet been explored. In this study, we used a mouse model of dextran sulfate sodium (DSS)-induced colitis and explored the effects of cucurbitacin IIb on colitis symptoms, inflammatory responses, microbiota, and metabolite profiles. The results showed that cucurbitacin IIb alleviated colitis symptoms including body weight loss, an increase in the disease activity index, and elevated levels of myeloperoxidase and eosinophil peroxidase content. Additionally, it ameliorated intestinal morphology impairment, reduced the phosphorylation of NFκB protein, and mitigated accumulation of pro-inflammatory cytokines IL-6 and IL-1β. Furthermore, cucurbitacin IIb alleviated alterations in gut microbial composition and metabolites in DSS-treated mice. However, antibiotic treatment diminishes the beneficial effects of cucurbitacin IIb on colitis. We further found that transplantation of fresh feces or heat-inactivated feces from mice treated with cucurbitacin IIb to DSS-treated mice alleviated colitis, similar to the effects of cucurbitacin IIb. Collectively, our results suggest that cucurbitacin IIb exerted anti-inflammatory effects in colitis by regulating the microbiota composition and metabolites, thereby alleviating colitis symptoms.

## 1 Introduction

Inflammatory bowel disease (IBD) is a chronic disorder characterized by recurrent episodes of intestinal inflammation. Although genetic susceptibility and environmental factors are considered major etiologies, the mechanisms underlying the pathogenesis of IBD remain to be fully elucidated. For decades, the rapidly increasing incidence of IBD worldwide has highlighted the urgent need to develop effective, efficient, and safe therapeutic approaches to combat the disease [1]. Gut microbes have been proposed as promising targets for the prevention and treatment of IBD because alterations in their composition and function play critical and central roles in disease pathogenesis [2,3]. Moreover, given that metabolites are the primary signals that mediate interactions between the gut microbiota and the host [4], they are considered key actors in IBD.

Cucurbitacins, members of the triterpenoid family found in Cucurbitaceae plants, have demonstrated a wide range of pharmacological activities. Hemsleyadine tablets, containing primarily cucurbitacin IIa and IIb, have shown therapeutic effects on intestinal diseases including enteritis and bacillary dysentery [5]. These findings indicate that cucurbitacins may have beneficial effects on inflammatory responses. Notably, cucurbitacin IIb exerted strong anti-inflammatory activity by preventing the nuclear translocation of p65 NF-κB in concanvalin A-activated lymphocytes [5]. Moreover, cucurbitacin IIb treatment increased the Treg cell percentage and decreased the Th17 cell percentage in mice with systemic lupus erythematosus [6]. Meanwhile, cucurbitacin IIb reduced the expression of IL-6 and IL-17 while enhancing the expression of IL-10 and TGF-β in the lymphocytes of mice with systemic lupus erythematosus. Moreover, cucurbitacin IIb regulated the IL-6 pathway in a mouse model of skeletal muscle atrophy [7].

The abovementioned reports suggest that cucurbitacin IIb could be a promising anti-inflammatory substance owing to its *in vitro* and *in vivo* inflammation-alleviating capability with fewer side effects and low toxicity [8]. However, its effects and mechanism of action in colitis remain unknown. Furthermore, the involvement of the gut microbiota remains to be explored. Exploring the effects of cucurbitacin IIb on gut microbes would expand our knowledge in the mechanism of cucurbitacin IIb on intestinal inflammation. Thus, in the present study, we explored the beneficial effects of cucurbitacin IIb on colitis symptoms, inflammatory response, and the composition of the gut microbiota and metabolites in mice treated with dextran sulfate sodium (DSS). Additionally, we studied whether the gut microbiota plays an indispensable role in the effects of cucurbitacin IIb on colitis by treating mice with antibiotics and fecal microbiota transplantation (FMT). The findings in the present study are expected to provide evidences for the application of cucurbitacin IIb in the treatment of colitis.

## 2 Materials and methods

### 2.1 Animals

Animal procedures were approved by the Protocol Management and Review Committee of Changsha Health Vocational College (No.2023006). All C57BL/6 male mice were purchased from SLAC Laboratory Animal (Changsha, China). After acclimated for one week in the pathogen-free colony with a temperature of 22 ± 2 °C and a relative humidity of 50 ± 5 %, all animals aged nine weeks were randomly allocated to treatment groups and free to access feed and water. All mice were anesthetized with 60 mg/kg sodium pentobarbital before sacrifice at the end of each experiments.

### 2.2 DSS-induced colitis

DSS with the molecular weight of 36, 000–50, 000 kDa was purchased from MP Biomedicals (Cat# 160110, Shanghai, China) and dissolved into autoclaved drinking water at the concentration of 2.5 % as previously did [9]. The mice were provided water containing DSS for 7 days. Body weight change was recorded and disease activity index (DAI) was scored by using a standard system (Supplementary Table 1).

### 2.3 Cucurbitacin IIb treatment

Twenty-one mice were assigned into three treatment groups (n = 7): CONT group, mice drinking autoclaved water; DSS, mice treated with water containing DSS from day 8 to day 14; EXT, mice treated with Cucurbitacin IIb from day 1 to day 14 and DSS from day 8 to day 14. Cucurbitacin IIb was purchased from Aladdin (Cat#C412645, Shanghai, China) and orally gavaged into mice with a concentration of 20 mg (dissolved in 0.1 mL PBS)/kg body weight once a day. This dose was chosen based on our preliminary experiments. On day 15, after fresh fecal samples were collected, all animals were used for blood collection from the retro-orbital sinus. Then, the mice were anesthetized and sacrificed by cervical dislocation for the determination of colon length and weight, and for the collection of samples of colonic tissue.

### 2.4 Determination of myeloperoxidase (MPO) and eosinophil peroxidase (EPO) content

ELISA kits purchased from Boyan Biological Technology (Nanjing, China) were used for the analysis of MPO (Cat#BY-EM230058) and EPO (Cat#BY-EM228249) contents in the distal colon according to the manufacturer's instructions.

### 2.5 Histological analyses

The distal colonic tissue was collected and immediately fixed with 4 % formaldehyde. Then, the samples were embedded in paraffin and cut into sections of 10-μm thickness. Furthermore, they were stained with hematoxylin and eosin (H&E) for morphology observation. The histological alteration of colitis was scored by using a standard system (Supplementary Table 2) as previously did [10].

### 2.6 Determination of inflammatory cytokine content

ELISA kits purchased from Boyan Biological Technology (Nanjing, China) were used for the analysis of IL-6 (Cat#BY-EM220188), IL-1β (Cat#BY-EM220174) and TNF-α (Cat#BY-EM220852) contents in the distal colon according to the manufacturer's instructions.

### 2.7 Western Blot analysis

Total proteins were extracted from distal colonic tissue using the protein extraction kit (Cat# P0013M, Beyotime, Shanghai, China). Samples of protein (20 μg) were separated by SDS-PAGE, and then transferred to PVDF membrane and blocked with skim milk for 2 h at 22 °C. The membranes were firstly incubated with primary antibodies against NFκB (Cat#Ab32536) and phosphorylated NFκB (Cat#Ab76302, Abcam, Shanghai, China) for 24 h at 4 °C and then incubated with secondary antibodies. After exposed to BeyoECL plus (Cat#P0018S, Beyotime), protein bands in the membranes were visualized.

### 2.8 Microbiota profiling

Fresh feces were collected for DNA extraction and then PCR reaction was performed with specific primers (341F: 5′-CCTAYGGGRBGCASCAG-3′ and 806R: 5′-GGACTACNNGGGTATCTAAT-3′) and Phusion High-Fidelity PCR Master Mix (Cat#E0553L, New England BioLabs, Beijing, China) for bacterial 16S rRNA gene sequences (V3+V4 region). Amplicons were used for library sequencing on an Illumina NovaSeq platform and 250 bp paired-end reads were generated. Sequence analysis were performed by Uparse software (Uparse v7.0.1001) and those with≥97 % similarity were assigned to the same operational taxonomic units (OTUs), which was used for taxonomy annotation using Silva 138.1. Alpha and beta diversity were calculated with QIIME software (Version 1.9.1) and displayed with R software (Version 2.15.3).

### 2.9 Untargeted metabolomics

Metabolites in the feces were extracted and then determined using a UHPLC-MS/MS system (ThermoFisher, Shanghai, China). The raw data files were processed using the Compound Discoverer (CD3.3, ThermoFisher) to obtain the normalized data. After matching with the mzCloud, mzVault and Masslist database, the qualitative and quantitative of the metabolites were further obtained. Then, all metabolites were annotated using the LIPIDMaps, HMDB and KEGG database. A univariate analysis was used to calculate the P-value. The metabolites with VIP>1 and P-value<0.05 and fold change >2 or <0.05 were considered to be significantly changed. The data was then normalized using z-scores and was plotted by Pheatmap package in R language for clustering heat maps. The correlation between differential metabolites and key bacteria were determined by Spearman correlation analyses.

### 2.10 Antibiotics and cucurbitacin IIb treatment experiments

Twenty-eight mice were assigned into four groups (n = 7): CONT group, mice drinking autoclaved water; ANT group, mice were orally gavaged with antibiotics mixture for 14 days; ANT + DSS group, mice were orally gavaged with antibiotics mixture for 14 days and treated with water containing 2.5 % DSS from day 8 to day 14; EXT + ANT + DSS group, mice were orally gavaged with antibiotics mixture and 20 mg cucurbitacin IIb/kg body weight from day 1 to day 14 and treated with water containing 2.5 % DSS from day 8 to day 14. The mice in the ANT, ANT + DSS and EXT + ANT + DSS group were orally gavaged with 200 μL antibiotics containing neomycin 1 mg/mL, vancomycin 0.5 mg/mL, ampicillin 1 mg/mL, metronidazole 1 mg/mL, and streptomycin 1 mg/mL (Sigma-Aldrich, Shanghai, China) daily for 14 consecutive days to eliminate intestinal bacteria. On day 15, all animals were sacrificed for sample collection.

### 2.11 Fecal microbiota transplantation (FMT) experiments

Twenty-one mice were assigned into three groups (n = 7): DSS group, control mice; FMT group, a total of 5 mg feces were diluted and suspended into 200 μL autoclaved drinking water, and then gavaged into the mice every other day; heat-inactivated FMT group, a total of 5 mg feces were firstly heated at 80 °C for 60 min [11], then diluted and suspended into 200 μL autoclaved drinking water, and gavaged into the mice every other day. Fresh feces were collected from mice treated with 20 mg cucurbitacin IIb/kg body weight daily for 14 consecutive days. The experiment lasted 14 days and all mice were treated with water containing 2.5 % DSS from day 8 to day 14. On day 15, all animals were sacrificed for sample collection.

### 2.12 Statistical analysis

All statistical analysis was performed with one-way ANOVA followed by Student-Newman-Keuls post hoc test using the SPSS Statistics 18.0 Software. All data was expressed as mean ± SEM and P value < 0.05 was considered to be significant.

## 3 Results

### 3.1 Cucurbitacin IIb alleviated DSS-induced colitis and inhibited NFκB signaling pathway

**Fig. 1:**
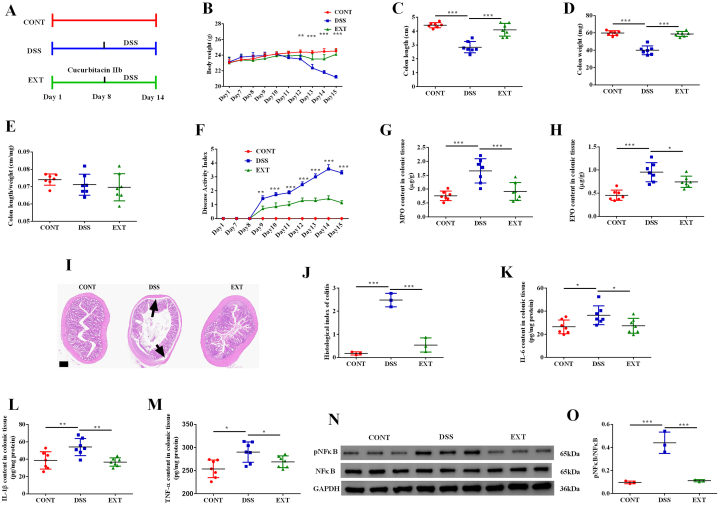
Cucurbitacin IIb alleviated DSS-induced colitis and inhibited NFκB signaling pathway. (A) The timeline and experiment design; (B) Body weight; (C) Colon length; (D) Colon weight; (E) Colon length/colon weight; (F) Disease activity index; (G) MPO content in colonic tissue; (H) EPO content in colonic tissue; (I) Colonic morphology; arrows, obvious edema; (J) Histological index of colitis; The content of IL-6 (K), IL-1β (L) and TNF-α (M) in colonic tissue; (N) Phosphorylated NFκB protein expression; (O) The ratio of phosphorylated NFκB to total NFκB protein. Data are expressed as the mean ± SEM, n = 7. ∗*P* < 0.05. ∗∗*P* < 0.01. ∗∗∗*P* < 0.001. MPO, myeloperoxidase; EPO, eosinophil peroxidase.

We firstly explored the effects of cucurbitacin IIb treatment on DSS-induced colitis (Fig. 1A). We found that cucurbitacin IIb prevented body weight loss and the decreases of colon length and weight caused by DSS treatment (Fig. 1B–D). No change in the ratio of colon length to weight was observed among the treatment groups (Fig. 1E). DSS caused a significant increase of DAI, while cucurbitacin IIb alleviated such change (Fig. 1F). Cucurbitacin IIb significantly decreased the content of MPO and EPO in the colonic tissue of DSS-treated mice (Fig. 1G–H). Cucurbitacin IIb alleviated DSS-induced impairment of colonic morphology indicated by obvious edema and significantly decreased histological index of colitis (Fig. 1I–J). Furthermore, cucurbitacin IIb decreased the content of IL-6, IL-1β and TNF-α in the colonic tissue (Fig. 1K–M), and inhibited the phosphorylation of NFκB protein (Fig. 1N–O).

### 3.2 Cucurbitacin IIb improved the alteration of microbiota composition caused by DSS

**Fig. 2:**
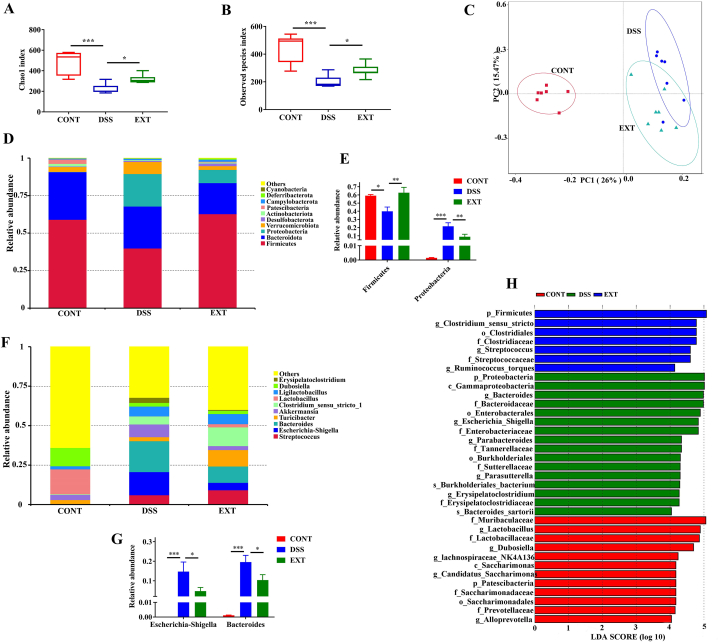
Cucurbitacin IIb alleviated the alteration of microbiota composition caused by DSS. (A) Chao1 index; (B) Observed species index; (C) The PCoA plot based on unweighted unifrac beta diversity analyses; (D) Relative abundance of predominant bacteria at the phylum level; (E) Relative abundance of Firmicutes and Proteobacteria; (F) Relative abundance of predominant bacteria at the genus level; (G) Relative abundance of *Escherichia-Shigella* and *Bacteroides*; (H) Bacterial biomarkers in the treatment groups (LDA score value > 4 or < −4). Data are expressed as the mean ± SEM, n = 7. ∗*P* < 0.05. ∗∗∗*P* < 0.001.

We then explored the effects of cucurbitacin IIb treatment on microbiota composition in DSS-treated mice. We firstly found that cucurbitacin IIb alleviated DSS-caused decrease of Chao1 and Observed species index of alpha diversity (Fig. 2A–B), while it did not affect Shannon and Simpson index (Supplementary Figs. 1 and 2). Based on unweighted unifrac beta diversity analyses, the PCoA plot showed apparent separation between microbiota in control mice and mice treated with DSS, while microbiota composition in mice treated with DSS and cucurbitacin IIb were also separated (Fig. 2C). DSS treatment significantly increased the relative abundance of the phylum Proteobacteria and decreased Firmicutes abundance, while cucurbitacin IIb alleviated these alterations (Fig. 2D–E). DSS treatment increased the relative abundance of the genus *Escherichia-Shigella* and *Bacteroides*, while cucurbitacin IIb decreased their abundances (Fig. 2F–G). Additionally, cucurbitacin IIb alleviated DSS-induced alteration of microbiota composition in the class, order and family levels (Supplementary Figure 3, 4 and 5). As shown by the results of LEfSe analysis, mice in the CONT group was mainly characterized with *Muribaculaceae* and *Lactobacillaceae*, mice in the DSS group was mainly characterized with Proteobacteria and Bacteriodaceae, while mice in the EXT group was mainly characterized with Firmicutes, Clostridiaceae and Streptococcaceae (Fig. 2H).

### 3.3 Cucurbitacin IIb improved the alteration of colonic metabolites caused by DSS

**Fig. 3:**
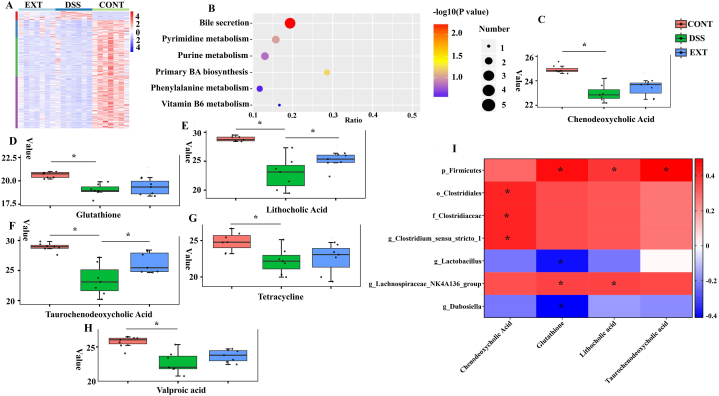
Cucurbitacin IIb alleviated the alteration of colonic metabolites caused by DSS. (A) Heatmap showing the alteration of metabolites; (B) KEGG analysis revealing the mainly affected pathways; Relative abundance of chenodeoxycholic acid (C), glutathione (D), lithocholic acid (E), taurochenodeoxycholic acid (F), tetracycline (G) and valproic acid (H); (I) Correlation of major bacteria abundance and metabolite level. n = 7. ∗*P* < 0.05.

We further explored the effects of cucurbitacin IIb treatment on the composition of colonic metabolites in DSS-treated mice. The heatmap showed that cucurbitacin IIb alleviated the alterations of many metabolites caused by DSS treatment (Fig. 3A). KEGG analysis revealed that bile secretion and primary bile acid biosynthesis were the mostly affected pathways (Fig. 3B). Metabolites including chenodeoxycholic acid, glutathione, lithocholic acid, taurochenodeoxycholic acid, tetracycline and valproic acid involved in these pathways, were significantly decreased by DSS treatment, while cucurbitacin IIb significantly increased lithocholic acid and taurochenodeoxycholic acid contents, and tended to increase chenodeoxycholic acid, glutathione, tetracycline and valproic acid contents (Fig. 3C–H). Furthermore, correlation analyses of metabolites and bacteria showed that differential metabolites were correlated with gut microbes (Fig. 3I). Notably, Firmicutes was positively correlated with glutathione, lithocholic acid and taurochenodeoxycholic acid; Clostridiales, Clostridiaceae and *Clostridium sensu stricto 1* were positively correlated with chenodeoxycholic acid; *Lactobacillus* and *Dubosiella* were negatively correlated with glutathione; *Lachnospriraceae Nk4A136* group was positively correlated with glutathione and lithocholic acid.

### 3.4 Antibiotics treatment diminished the beneficial effects of cucurbitacin IIb on colitis

**Fig. 4:**
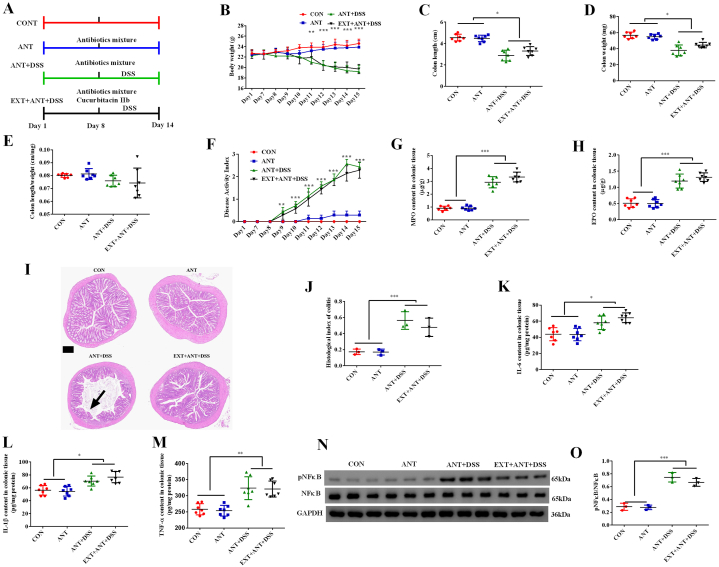
Antibiotics treatment diminished the beneficial effects of cucurbitacin IIb on colitis. (A) The timeline and experiment design; (B) Body weight; (C) Colon length; (D) Colon weight; (E) Colon length/colon weight; (F) Disease activity index; (G) MPO content in colonic tissue; (H) EPO content in colonic tissue; (I) Colonic morphology; arrows, irregularly aligned villus; (J) Histological index of colitis; The content of IL-6 (K), IL-1β (L) and TNF-α (M) in colonic tissue; (N) Phosphorylated NFκB protein expression; (O) The ratio of phosphorylated NFκB to total NFκB protein. Data are expressed as the mean ± SEM, n = 7. ∗*P* < 0.05. ∗∗*P* < 0.01. ∗∗∗*P* < 0.001. MPO, myeloperoxidase; EPO, eosinophil peroxidase.

We then explored whether diminishing gut bacteria by antibiotics affected the preventing effects of cucurbitacin IIb on colitis (Fig. 4A). The results showed that antibiotics treatment had no effects on body weight, colon weight and length, while cucurbitacin IIb did not alleviate body weight loss, and the decreases of colon length and weight caused by DSS treatment in those mice treated with antibiotics (Fig. 4B–D). No change in the ratio of colon length to weight was observed among the treatment groups (Fig. 4E). Cucurbitacin IIb did not affect DAI in those mice treated with antibiotics (Fig. 4F). Cucurbitacin IIb also did not affect the content of colonic MPO and EPO (Fig. 4G–H), impairment of colonic morphology (Fig. 4I) and histological index of colitis (Fig. 4J) in those mice treated with antibiotics. Furthermore, cucurbitacin IIb did not decrease the content of colonic IL-6, IL-1β and TNF-α (Fig. 4K–M), and inhibited the phosphorylation of NFκB protein (Fig. 4N–O) in those mice treated with antibiotics.

### 3.5 FMT alleviated colitis as cucurbitacin IIb did

**Fig. 5:**
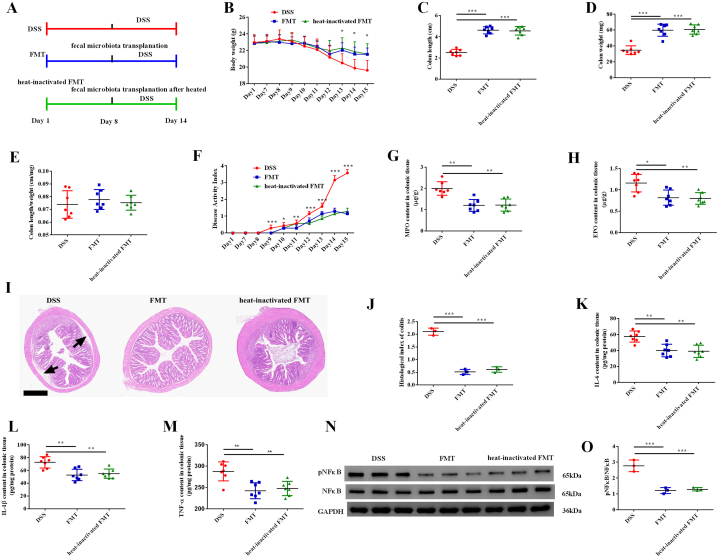
Fecal microbiota transplantation alleviated colitis. (A) The timeline and experiment design; (B) Body weight; (C) Colon length; (D) Colon weight; (E) Colon length/colon weight; (F) Disease activity index; (G) MPO content in colonic tissue; (H) EPO content in colonic tissue; (I) Colonic morphology; arrows, obvious edema and irregularly aligned villus; (J) Histological index of colitis; The content of IL-6 (K), IL-1β (L) and TNF-α (M) in colonic tissue; (N) Phosphorylated NFκB protein expression; (O) The ratio of phosphorylated NFκB to total NFκB protein. Data are expressed as the mean ± SEM, n = 7. ∗*P* < 0.05. ∗∗*P* < 0.01. ∗∗∗*P* < 0.001. MPO, myeloperoxidase; EPO, eosinophil peroxidase.

Finally, we performed FMT experiment to explore whether gut microbiota and metabolites mediated the preventing effects of cucurbitacin IIb on colitis (Fig. 5A). We found that transplanted feces from mice treated with cucurbitacin IIb to DSS-treated mice could prevent body weight loss and the decreases of colon length and weight (Fig. 5B–D). Moreover, heat-inactivated feces could exert the same effects. No change in the ratio of colon length to weight was observed among the treatment groups (Fig. 5E). Both fresh feces and heat-inactivated feces could alleviate DAI (Fig. 5F), the increased content of colonic MPO and EPO (Fig. 5G–H), impairment of colonic morphology (Fig. 5I) and histological index of colitis (Fig. 5J) caused by DSS treatment. Furthermore, FMT and heat-inactivated FMT both decreased the content of colonic IL-6, IL-1β and TNF-α (Fig. 5K–M), and inhibited the phosphorylation of NFκB protein (Fig. 5N–O).

## 4 Discussion

Cucurbitacin IIb, as a member of cucurbitacins, play strong activities including anti-cancer, anti-inflammation and anti-proliferation. Torres-Moreno et al. suggested that cucurbitacin IIb from *Ibervillea sonorae* exerted pronounced effects on apoptosis and proliferation of tumor cells, indicating its anti-cancer ability [12]. Wang et al. reported that cucurbitacin IIb exerted strong anti-inflammatory effects via regulating several signaling pathways [5]. Additionally, several studies demonstrated that cucurbitacin IIb exhibited anti-proliferative effects and regulated cell cycle arrest via modulating STAT3 and EGFR/MAPK singling pathways in different cells [[12], [13], [14]]. These results also suggested that cucurbitacin IIb was less cytotoxic to non-cancer cells. Cucurbitacin IIb could target the PI3K/Akt signaling pathway [15], which is involved in the regulation of inflammation response [16]. Thus, cucurbitacin IIb could be a promising agent that used for the treatment of inflammation-related diseases [17], considering that it is an extract of natural plants. However, most of the previous studies explored the advantageous effects of cucurbitacin IIb *in vitro*. We in the present study demonstrated that cucurbitacin IIb alleviated DAI and inflammatory response in an animal model of colitis. More importantly, we suggested that gut microbes and metabolites could be key targets of cucurbitacin IIb and mediate its advantageous effects.

DSS-induced mice colitis is one of the widely used model of gastroenterology inflammation to explore the modulatory mechanisms of functional agents [18]. The colitis is majorly characterized by intestinal barrier dysfunction and over-accumulation of inflammatory cytokines [19]. We firstly found that cucurbitacin IIb reduced the histological index of colitis and alleviated the impairment of colonic morphology. Then, we focused on the changes of inflammation response since it is suggested that pro-inflammatory cytokines exacerbated barrier dysfunction [20]. We found that colonic MPO and EPO content were increased, which indicated infiltration of polymorphonuclear leukocytes and eosinophils [21], two of the representative histological characters of inflammation [22]. The exacerbated inflammation response was further confirmed by enhanced contents of IL-6 and IL-1β in the colonic tissue. Importantly, we found that the phosphorylation of NF-κB protein was reduced, indicated that this signaling pathway might be one of the major targets of cucurbitacin IIb. Thus, our results suggested that cucurbitacin IIb might alleviate colitis mainly through modulating inflammatory response.

The intestinal tract is home to a complex and abundant aggregation of microorganisms. The gut microbes have a variety of physiological functions related to nutritional composition, immune function, and defense capability of the host. Dietary changes and other factors could cause maladaptive shifts in the composition and functions of gut microbes, as well as products of their metabolism [2]. Notably, increasing studies demonstrated that dysbiosis play a critical role in the development of IBD [23,24]. In the present study, we confirmed that dysbiosis occurred during the pathogenesis of IBD while cucurbitacin IIb alleviated the alterations of gut microbes and metabolites. Notably, a remarkable increase of *Escherichia-Shigella* was observed in DSS-treated mice while its abundance was significantly decreased by cucurbitacin IIb. It is reported that about 10–15 % of individuals with IBD has a high abundance of *Escherichia-Shigella*, which is closely associated intestinal inflammation [25,26]. Additionally, cucurbitacin IIb increased the abundance of *Lactobacillus*, which exerts beneficial effects on intestinal health, although it is a minor member of the colonic microbiota [27]. Thus, these results indicated that cucurbitacin IIb could benefit gut microbiota composition and alleviate colitis. However, fewer study reported the effects of cucurbitacin IIb on gut microbes except that a previous study found that administration of *Cucumis melo* rich in cucurbitacins could change the composition of gut microbiota [28]. Bile acids are critically involved in the pathogenesis of IBD and regulation of bile acid homeostasis help prevent the development of colitis. Notably, an accumulation of bile acids was also reported in the colonic tissue of DSS-treated mice [29]. However, we found that several secondary bile acids including chenodeoxycholic acid, lithocholic acid, and taurochenodeoxycholic acid were decreased in the feces of mice with colitis. Cucurbitacin IIb could remarkably increase the content of taurochenodeoxycholic acid and lithocholic acid, which have been reported to reduce gut inflammation [30]. Importantly, both taurochenodeoxycholic acid and lithocholic acid were positively correlated with the Firmicutes phylum, whose abundance was decreased by DSS while increased by cucurbitacin IIb treatment. The abundance of Firmicutes is related to plasma levels of bile acids [31] and *Lactobacillus* alleviates inflammatory response through modulating bile acid metabolism [32]. Additionally, dietary *Lactobacillus* results in alteration of *Escherichia-Shigella* abundance in association with intestinal bile acid metabolism [33]. These findings are in line with our results, which indicated that close relationships between these abovementioned microbes and bile acid metabolism. However, no specific relationship between bile acids including taurochenodeoxycholic acid and lithocholic acid and gut microbes including *Lactobacillus* and *Escherichia-Shigella* were reported as we known.

Although our results suggested that cucurbitacin IIb could alleviate colitis via regulating gut microbial composition and metabolites, more studies should be performed to demonstrate the specific mechanisms, which could promote the use of cucurbitacin IIb in the treatment of colitis. Specifically, the effects of cucurbitacin IIb on gut microbiota composition and function remains to be largely unknown. Future study should focus on how does cucurbitacin IIb affect the composition of gut microbes and which microbes are the primary targets of cucurbitacin IIb. Additionally, we in the present study found that the metabolites were also affected by cucurbitacin IIb and their alterations were significantly correlated with the abundance of certain gut microbes. However, whether these metabolites were the key factors that mediates the beneficial effects of cucurbitacin IIb on colitis need to be further elucidated. Moreover, increasing studies demonstrated that extracellular vesicles are potential mediators during host-microbe interaction [34,35]. Thus, it is worth noting that whether cucurbitacin IIb could affect the production of microbe- and host-derived extracellular vesicles and whether extracellular vesicles mediate the effects of cucurbitacin IIb.

In conclusion, our results suggested that cucurbitacin IIb could alleviate colitis and the alterations of gut microbial composition and metabolites. However, antibiotics treatment diminished the beneficial effects of cucurbitacin IIb on colitis. Meanwhile, the results showed that transplantation of either fresh feces from mice treated with cucurbitacin IIb or heat-inactivated feces to DSS-treated mice alleviated colitis as cucurbitacin IIb did. Collectively, our results suggested that cucurbitacin IIb exerted its anti-inflammatory effects in colitis through regulating microbiota composition and metabolites, leading to the alleviation of colitis symptoms.

## Funding

This work was supported by the Doctoral Development Foundation of LiHuiLi Hospital (No. 2023BSKY-ZYY).

## Ethics statement

Animal procedures were approved by the Protocol Management and Review Committee of Changsha Health Vocational College (No.2023006).

## Data availability statement

The 16S rRNA gene sequence data have been deposited in the NCBI BioProject database (https://www.ncbi.nlm.nih.gov/bioproject/) under accession numbers PRJNA1079036.

## CRediT authorship contribution statement

**Yinyin Zhao:** Writing – original draft, Methodology, Investigation, Funding acquisition, Data curation, Conceptualization. **Kangxiao Guo:** Writing – original draft, Methodology, Investigation, Formal analysis, Data curation. **Yongwang Yan:** Writing – review & editing, Validation, Supervision, Conceptualization. **Binyuan Jiang:** Writing – review & editing, Validation, Supervision, Conceptualization.

## Declaration of competing interest

The authors declare that they have no known competing financial interests or personal relationships that could have appeared to influence the work reported in this paper.
