# Supplementary material for: Cucurbitacin IIb alleviates colitis via regulating gut microbial composition and metabolites
Source: Heliyon. 2024 Sep 18;10(18):e38051. doi: 10.1016/j.heliyon.2024.e38051 (PMC11437856; doi:10.1016/j.heliyon.2024.e38051)

**Supplemental Table 1 Assessment of Disease Activity**

The disease activity index (DAI) based on body weight change, stool consistency and rectal bleeding, of the animal during the experiment was calculated according to a standard scoring system.

| Scores | Body weight change | Stool consistency | Rectal bleeding |
| --- | --- | --- | --- |
| 0 | No weight loss | Normal | Negative hemoccult |
| 1 | Weight loss of 0.1-5% | Soft but still formed | Positive hemoccult |
| 2 | Weight loss of 5-10% | Very soft | Blood traces in stool visible |
| 3 | Weight loss >10% | Diarrhea | Rectal bleeding |

**Supplemental Table 2 Histological grading of colitis**

| Feature | Score | Description |
| --- | --- | --- |
| Damage | 0 | None |
|  | 1 | Loss of the basal 1/3 of the crypt |
|  | 2 | Loss of the basal 2/3 of the crypt |
|  | 3 | Loss of entire crypt but intact surface |
|  | 4 | Loss of entire crypt and the surface |

**Supplemental Figure 1 Shannon index of microbiota composition**


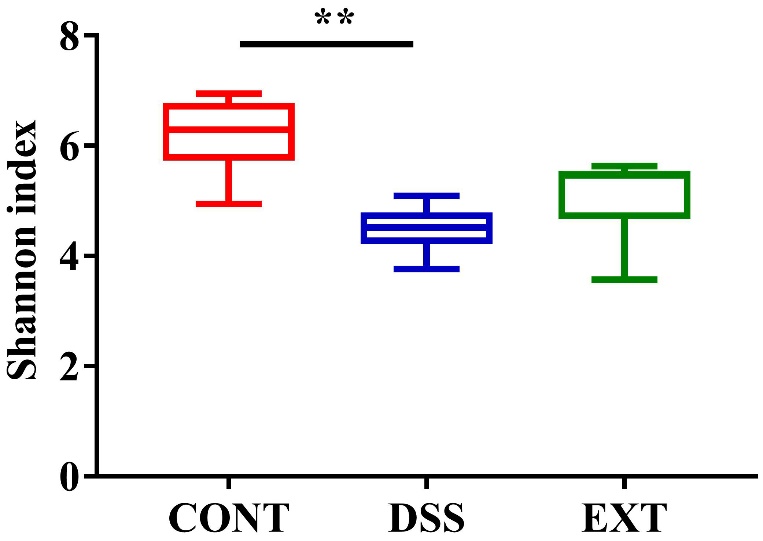


Data are expressed as the mean ± SEM, n=7. **P < 0.01.

**Supplemental Figure 2 Simpson index of microbiota composition**


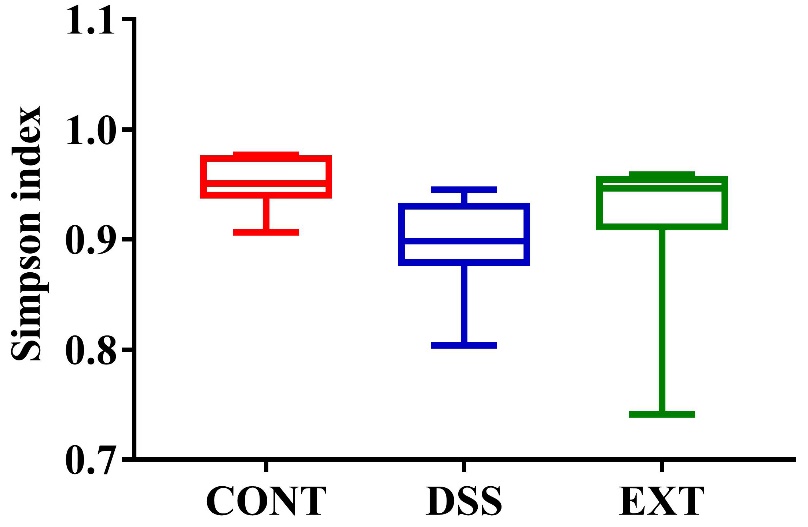


Data are expressed as the mean ± SEM, n=7.

**Supplemental Figure 3 Relative abundance of predominant bacteria at the class level**


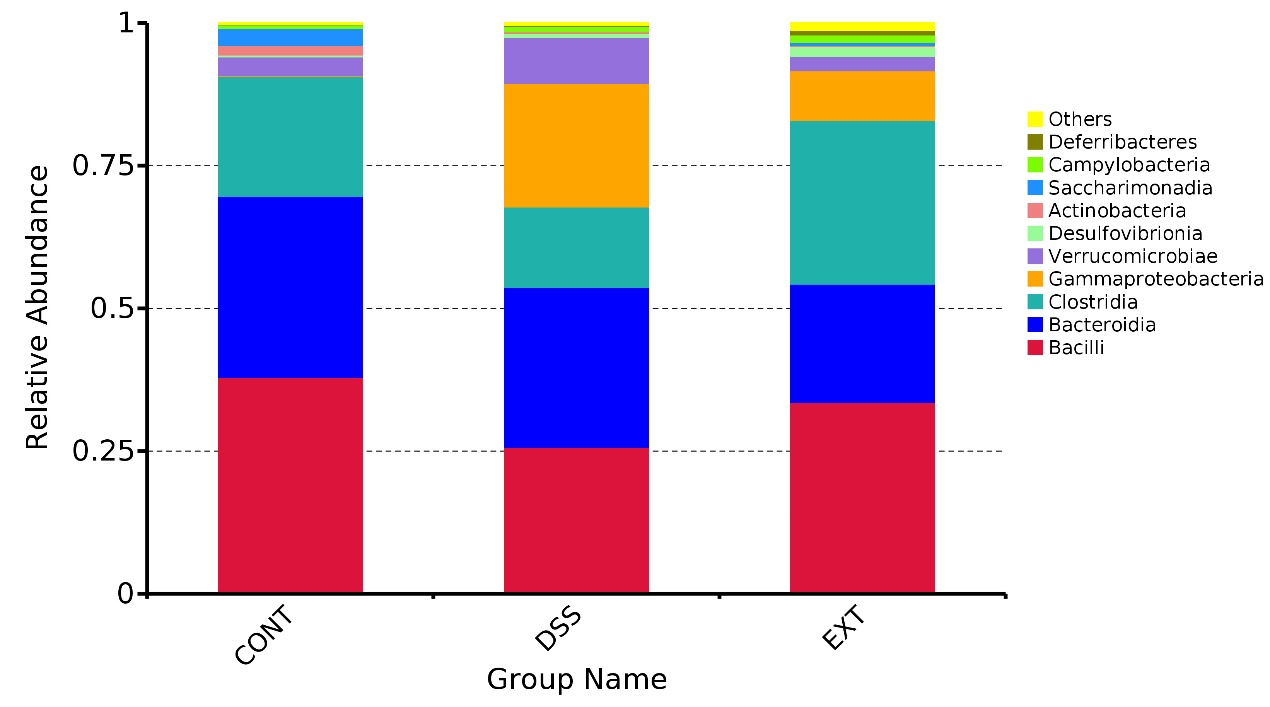


**Supplemental Figure 4 Relative abundance of predominant bacteria at the order level**


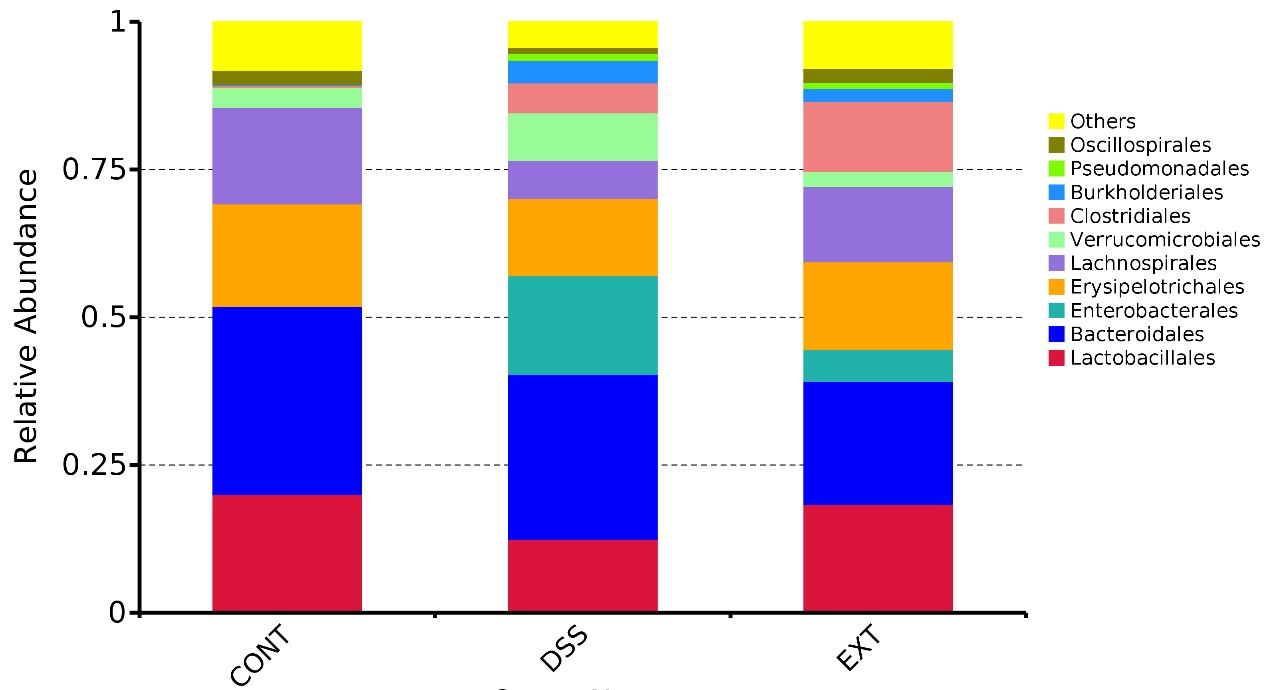


**Supplemental Figure 5 Relative abundance of predominant bacteria at the family level**


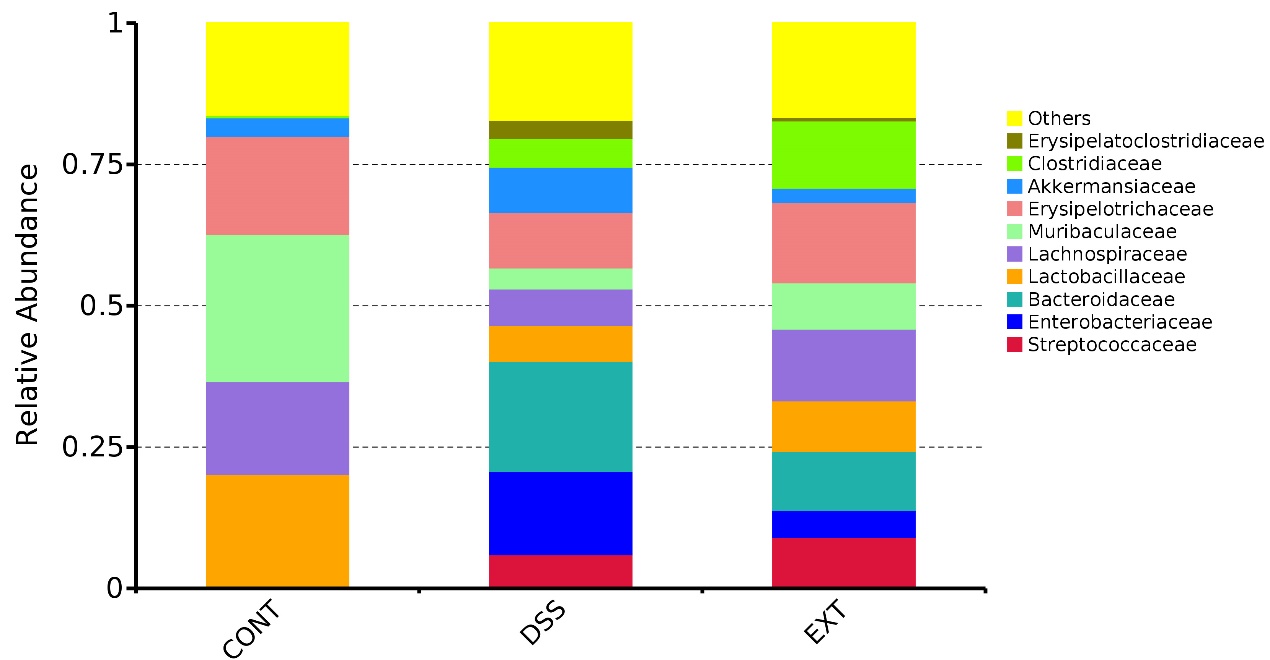


**Uncropped versions of figures 1N**

GAPDH


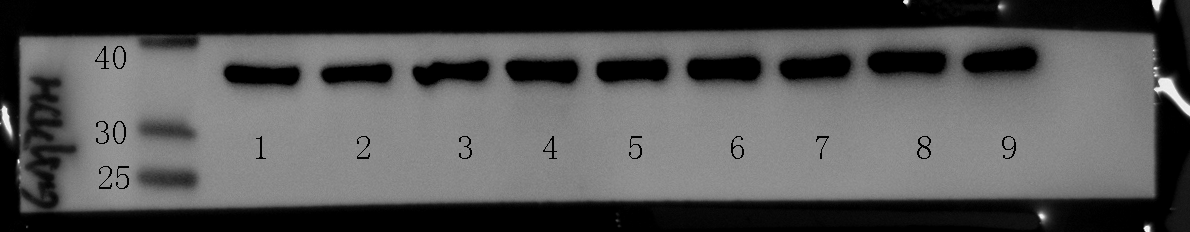


NFκB


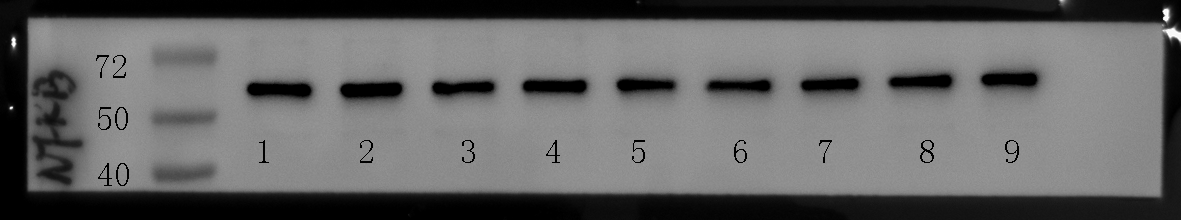


pNFκB


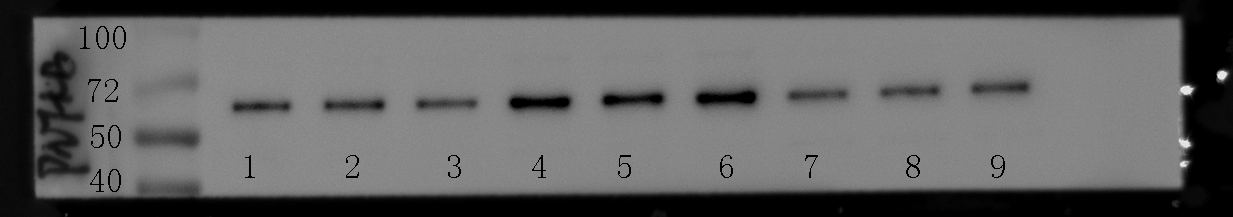


**Uncropped versions of figures 4N**

GAPDH


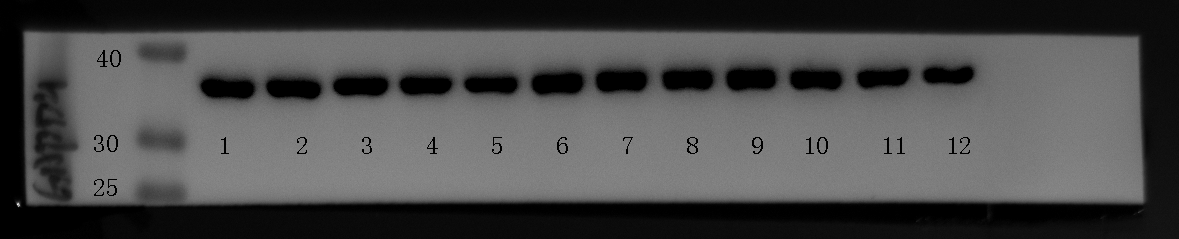


NFκB


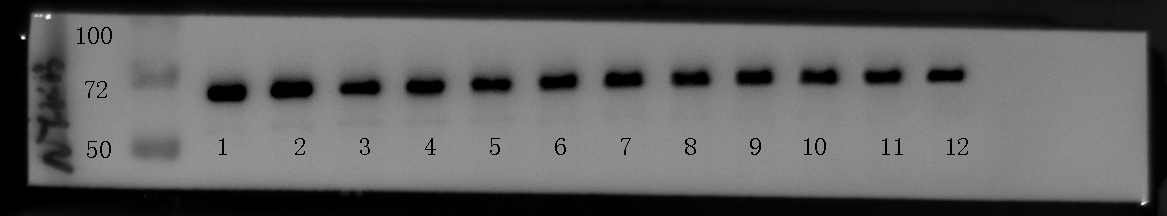


pNFκB


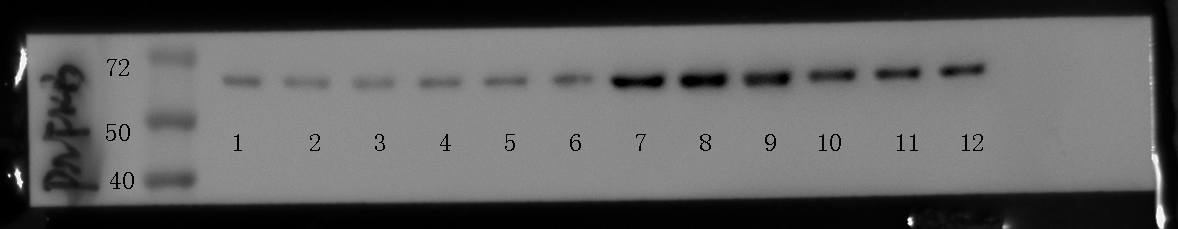


**Uncropped versions of figures 5N**

GAPDH


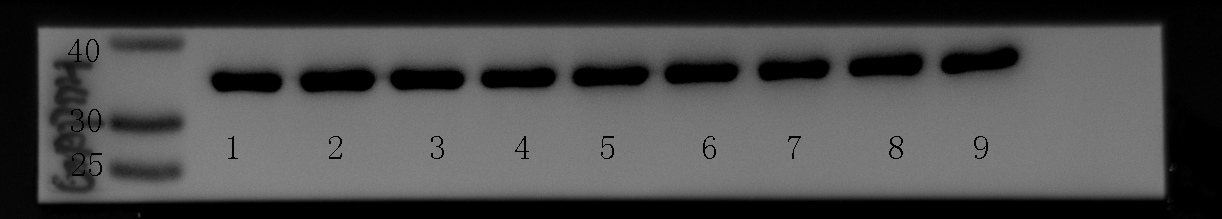


NFκB


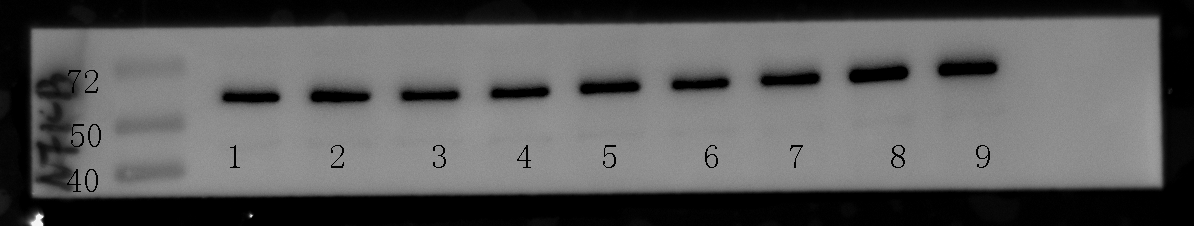


pNFκB


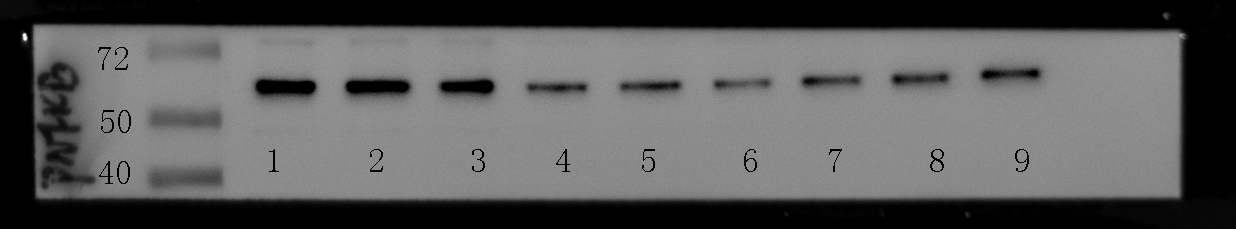

Supplement: Multimedia component 1 [file mmc1.docx]
